# Temporal virtual reality-guided, dual-task, trunk balance training in a sitting position improves persistent postural-perceptual dizziness: proof of concept

**DOI:** 10.1186/s12984-022-01068-6

**Published:** 2022-08-20

**Authors:** Tomoya Yamaguchi, Toru Miwa, Kaoru Tamura, Fumiko Inoue, Naomi Umezawa, Toshiki Maetani, Masahiko Hara, Shin-ichi Kanemaru

**Affiliations:** 1 Department of Otolaryngology-Head and Neck Surgery, Tazuke Kofukai Medical Research Institute, Kitano Hospital, Osaka, Japan; 2 Department of Otolaryngology-Head and Neck Surgery, Kyoto University, Kyoto, Japan; 3 Department of Otolaryngology, Osaka Metropolitan University School of Medicine, 1-4-3 Asahi-machi, Abeno-ku, Osaka, 545-8585 Japan; 4 Department of Medical Device Development, mediVR, Inc., Toyonaka, Japan; 5 Center for Community-Based Healthcare Research and Education, Shimane University, Izumo, Japan

**Keywords:** Virtual reality, Persistent postural-perceptual dizziness, Rehabilitation

## Abstract

**Background:**

Persistent postural-perceptual dizziness (PPPD) is a newly defined disorder characterized by functional dizziness. Due to its recent discovery, definitive treatment for PPPD has not been established; therefore, this study aimed to assess the effectiveness of virtual reality (VR)-guided, dual-task, trunk balance training for the management of PPPD using the mediVR KAGURA system.

**Methods:**

We analyzed data of patients who presented with PPPD from January 1, 2021, to February 28, 2021. The VR group included patients who underwent mediVR KAGURA-guided training for 100 tasks (10 min). Patients with PPPD who received standard treatment and rehabilitation were assigned to the control group. Equilibrium tests were performed at baseline and immediately after mediVR KAGURA-guided training to examine its effectiveness in improving static and dynamic balance. Additionally, clinical questionnaires related to balance disorders were administered at baseline and 1 week after mediVR KAGURA-guided training to examine its effects on balance-related symptoms. The primary outcome was improvements in static and dynamic balance and Niigata PPPD Questionnaire (NPQ) scores.

**Results:**

VR-guided training using mediVR KAGURA improved objective outcomes, including static and dynamic postural stability, after a single 10-min training session. Additionally, mediVR KAGURA-guided training improved scores on the Hospital Anxiety and Depression Scale and NPQ 1 week after the 10-min training session.

**Conclusion:**

VR-guided training using mediVR KAGURA represents a viable method for managing balancing ability, anxiety, and symptoms in patients with PPPD. Such training provides a safe and cost-effective solution for PPPD management. Further studies are required to evaluate the clinical efficacy of this strategy.

*Trial registration*: Institutional Ethics Committee of Kitano Hospital, approval number: 1911003. Registered 18 December 2019, https://kitano.bvits.com/rinri/publish_document.aspx?ID=426.

**Supplementary Information:**

The online version contains supplementary material available at 10.1186/s12984-022-01068-6.

## Background

Persistent postural-perceptual dizziness (PPPD) is a functional dizziness disorder characterized by a chronic vestibular syndrome lasting for > 3 months, typically preceded by acute vestibular symptoms [1]. According to the International Classification of Diseases 11th edition, PPPD was previously referred to as phobic postural vertigo (PPV) or chronic subjective dizziness (CSD) [2]. PPPD presents as core vestibular symptoms including dizziness, unsteadiness, or non-spinning vertigo, and it is exacerbated during upright posture/walking, active or passive movement, or exposure to complex visual stimuli [2]. The disorder is caused by long-term maladaptation to a neuro-otological, medical, or psychological event that triggered vestibular symptoms. Additionally, it is considered within the spectrum of other functional neurological disorders.

The core dogma that explains PPPD is “sensory weighting” [1, 3–9]. The postural control system involves a complex organization of visual, vestibular, and somatosensory inputs, which are relayed to the musculoskeletal system by the central nervous system [10]. Normal postural stability occurs due to a balance among the “weights” of these three types of sensory input and accurate processing of signals from the central nervous system to motile organs. In patients with PPPD, the processing of spatial orientation information shifts to favor visual over vestibular and somatosensory inputs, and there is a failure of higher-order cortical mechanisms to modulate visual, vestibular, and somatosensory inputs [1, 4–9]. Thus, patients with PPPD exhibit altered and disorganized “sensory weighting” in the central nervous system [1, 3–9]. An intractable and persistent disease usually implies a higher degree of maladaptation, more severe disability, and more ingrained illness beliefs. Although previous studies have suggested the long-term benefits of early treatment for PPV and CSD [11, 12], definitive treatment for PPPD based on randomized controlled trials has yet to be approved. However, numerous studies have investigated therapeutic strategies for managing PPPD, including selective serotonin reuptake inhibitors and serotonin-norepinephrine reuptake inhibitors [1, 13], vestibular rehabilitation [13–15], cognitive-behavioral therapy [16–18], and electrical stimulation [19, 20].

Virtual reality (VR) technology has been recently introduced in a variety of clinical settings, including physical, occupational, cognitive, and psychological rehabilitation or training [21, 22]. In particular, physicians and therapists have expressed interest in the application of VR technology to balance training in patients with neurological disorders such as stroke [21, 22]. Research has indicated that this technology can be applied for post-hospitalization rehabilitation in patients with walking disabilities, particularly older adults presenting with disuse syndrome [23]. VR technology also has the potential to overcome the scarcity of human and economic resources in rehabilitation medicine [24]; however, the clinical efficacy of VR rehabilitation relative to traditional methods remains unclear [21, 22, 25, 26]. Although a few reports regarding VR rehabilitation for walking or balance training using a Wii Fit system (Nintendo, Kyoto, Japan) have been published, none have validated whether the use of VR technology can improve walking or balance in patients with chronic vestibular disorders [27, 28]. A recent study reported successful management of muscle atrophy or cerebellar ataxia resistant to traditional therapeutic management using mediVR KAGURA-guided, dual-task balance training (mediVR KAGURA, mediVR, Inc., Toyonaka, Japan, commercially available since February 4, 2019) [29, 30]. The mediVR KAGURA system was developed to help patients regain their walking ability by improving dual-task processing and trunk balance [29, 30].

In the present study, we investigated the effectiveness of VR-guided, dual-task trunk balance training using the mediVR KAGURA system for the management of PPPD.

## Methods

### Participants

Patients with PPPD at our hospital from January 1, 2021, to February 28, 2021, were recruited for the current study. Inclusion criteria were as follows: (i) age between 20 and 100 years; (ii) score of > 27 on the Niigata PPPD Questionnaire (NPQ), as described in Additional file 1 [31]; (iii) visit to the hospital more than twice between January 1, 2020, and December 31, 2020. Patients who presented with comorbidities (asthma and uncontrolled cardiac, pulmonary, gastrointestinal, renal, hepatic, endocrine, musculoskeletal, or oncological disorders) that would prevent them from completing the study were excluded. Clinical diagnosis of vestibular disorders that preceded PPPD was based on the diagnostic criteria published by the Japan Society for Equilibrium Research [32, 33] and the Bárány Society [2].

### Study design and outcome measures

We randomly divided the patients into two groups: the VR-guided training group (VR group) and the conventional physiotherapy group (control group). Patients in the VR group underwent VR-guided training using mediVR KAGURA for 100 tasks, which took approximately 10 min. In the VR group, equilibrium tests were performed at baseline and immediately after mediVR KAGURA-guided training to examine its effects on static and dynamic balance. In the control group, equilibrium tests were performed at baseline and 10 min after the physiotherapist performed conventional physiotherapy related to vestibular compensation via the vestibulo-ocular reflex. Additional assessments using clinical questionnaires evaluating vestibular disorders were performed at baseline and 1 week after mediVR KAGURA-guided training or conventional physiotherapy to examine the effects on symptoms related to vestibular disorders. All evaluations were blinded. All patients were treated with anti-vertigo/dizziness medications; other medications administered for comorbidities were not limited. Additionally, all patients were instructed to perform vestibular rehabilitation three times per day at home to acquire vestibular compensation via the vestibulo-ocular reflex.

The primary outcome was the effect of VR on static and dynamic trunk balance and NPQ scores in patients with PPPD.

### Detailed methods for VR training

**Fig. 1:**
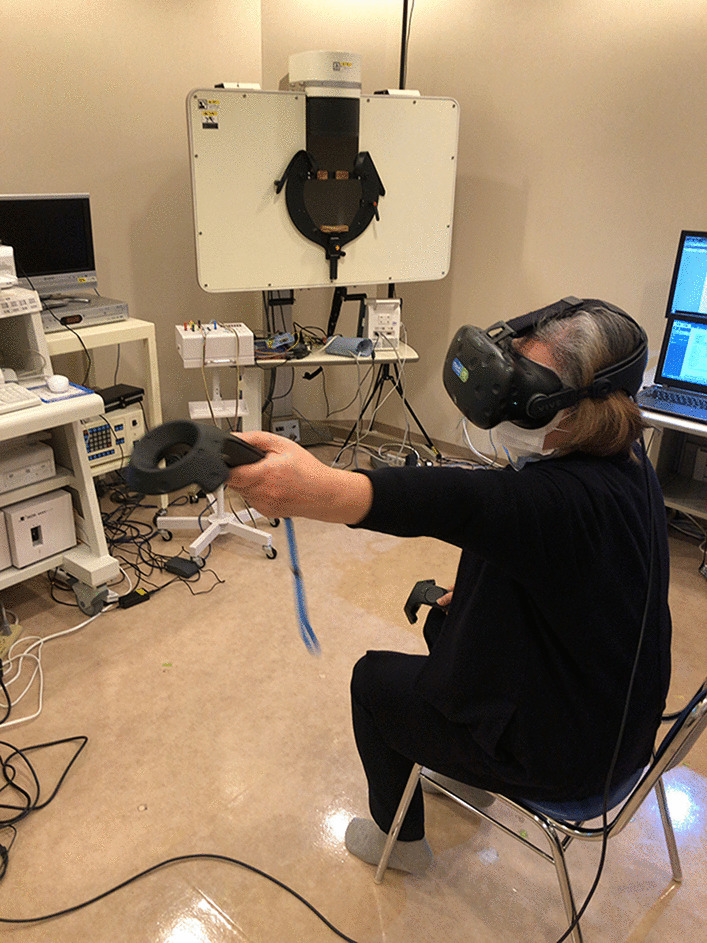
Image during VR training. *VR* virtual reality

In the 3D virtual space, the participants were instructed to catch falling red or blue objects or touch fixed red or blue targets with their right- or left-hand controllers, respectively. The training was performed with the patient in a sitting position to ensure safety, as training while standing is prohibited according to the manufacturer’s instructions. The mediVR KAGURA system provides a video game-like training environment to enhance motivation and ensure adherence. The therapist can help patients undergo training in a dual-task fashion, utilizing various levels of physical and cognitive stimulation through seven parameters. These parameters include the distance, angle, height, and size of the object; size of the controller; inter-task interval; and falling speed or time limit for each task (Fig. 1; Additional file 2). In addition, the therapist is permitted to encourage patients to decrease their tension and promote effective training.

Cognitive stimulation was designed to emulate how cognitive and attention functions are processed during each activity. For example, participants were instructed to catch red objects using the right-hand controller and blue objects using the left-hand controller. Differentiation of object colors emulates the cognitive processing required to differentiate traffic signals. Considering the seven parameters, recognizing a new object in 3D space and reaching a response in a timely manner emulates the cognitive processing involved in hazard perception and facilitates hazard avoidance in daily life (Fig. 1; Additional file 2).

The VR system places a radar screen at the center of the head-mounted display to indicate the direction of the next task, and the patient must refer to this radar screen regularly. Some gaming characteristics have been added to divert attention away from the radar screen while evaluating or training the patient’s attentiveness.

### Detailed assessment methods

Methodological details and criteria regarding the questionnaires and equilibrium tests are described in Additional file 1. The clinical questionnaires administered included the NPQ [31], the Dizziness Handicap Inventory (DHI) [34, 35], the Hospital Anxiety and Depression Scale (HADS) [36, 37], orthostatic dysregulation (OD) questionnaires [38, 39], Graybiel’s motion sickness test [40], and the Pittsburgh Sleep Quality Index (PSQI) [41, 42].

Equilibrium tests included stabilometry with or without foam posturography to assess steady-state postural control [43], while the Foulage stepping test was used to assess dynamic postural control. During stabilometry, the patients were instructed to stand on a strain-gauge force platform (GP-5000 stabilometer; Anima, Tokyo, Japan) for 60 s with their eyes open or closed and with or without foam rubber [44]. As described above, the postural control system involves the complex organization of visual, vestibular, and somatosensory inputs. Foam rubber posturography can assess sensory “dependence” or “weighting” among these three types of input [44]. When patients stand on a foam rubber force platform with their eyes closed, somatosensation from the foot and visual stimuli is reduced, which results in altered sensory weighting of vestibular inputs. Thus, this setup can indirectly reflect the function of the peripheral and central vestibular systems due to reductions in visual and somatosensory input [44]. To assess vestibular weighting, the velocity of the center of pressure (COP) was calculated in the eyes closed/foam rubber conditions (VCF; lower values indicate improvement in PPPD). Studies have indicated that Romberg’s ratio, which compares postural sway in eyes-open and eyes-closed conditions involving a foam rubber platform, may reflect visual dependence in a standing posture [44]. To assess visual weighting, the velocity of the Romberg’s ratio was calculated (VRF; lower values indicate improvement in PPPD). To assess somatosensory weighting, the velocity of the foam ratios (ratio of a measured parameter with or without the foam rubber platform) was calculated in the eyes-closed position (VFCF; higher values indicate improvement in PPPD). Measurements were performed under background noise conditions (approximately 50 dB).

Dynamic equilibrium was assessed using the Foulage test (FT) [45–47], which involves a quantified stepping test regulated at 120 bpm using a metronome. In this test, the patient is instructed to stand upright with both arms at the sides of the body and with closed feet, with the toes continuously touching the plate, allowing for alternating changes in the height of the heel only (rise of approximately 2–6 cm) [45, 46, 48]. Dynamic Romberg’s ratios (eyes-closed /eyes-open FT value; area of the front–back width of the locus) were assessed, with lower values indicative of PPPD improvement [45, 46, 48].

### Statistical analyses

Power and sample size calculations were conducted using the PS software (Ver. 3.1.6, Vanderbilt University, Nashville, TN, USA) [49]. This study aimed to include at least 10 patients to yield a power ≥ 0.80, assuming a medium effect size (i.e., d = 0.80 [50]) and an α ≤ 0.05. All statistical analyses were performed using GraphPad Prism version 8.0.0 for Windows, GraphPad Software, San Diego, California USA (www.graphpad.com). An unpaired t-test and Fisher’s exact test were used to compare age and sex between the groups, respectively. Parametric data related to questionnaire and equilibrium test results before and after training were assessed using paired t-tests, while non-parametric data related to these results were assessed using Mann–Whitney U-tests. Residual plots were used to confirm the accuracy of the assumptions made for outcomes. Statistical significance was set at *P* < 0.05. Evaluations were determined as “not applicable” if the calculated sample size after data collection was found to be insufficient for statistical analysis.

## Results

### Participants

Fourteen patients underwent mediVR KAGURA-guided training; however, two patients did not consent to share their data. Therefore, twelve patients (3 men, 9 women; mean age ± standard deviation (SD): 58.0 ± 17.1 years) were included in the VR group. The control group included 14 patients (3 men, 11 women; mean age ± SD: 63.5 ± 15.9 years). Detailed participant information is shown in Table 1.

**Table 1:** Patient characteristics

	VR group (n = 12)	Control group (n = 14)	P-value
Age	58.0 ± 17.1	63.5 ± 15.9	0.12^a^
Sex (M:F)	3:9	3:11	0.67^b^
Preceding vestibular disease	Meniere’s disease: 11 BPPV: 1	Meniere’s disease: 12 BPPV: 2	NA

*VR* virtual reality, *BPPV* benign paroxysmal positional vertigo

^a^Unpaired t-test

^b^Fisher’s exact test

### Static postural stability was improved by mediVR KAGURA-guided training in patients with PPPD

**Fig. 2:**
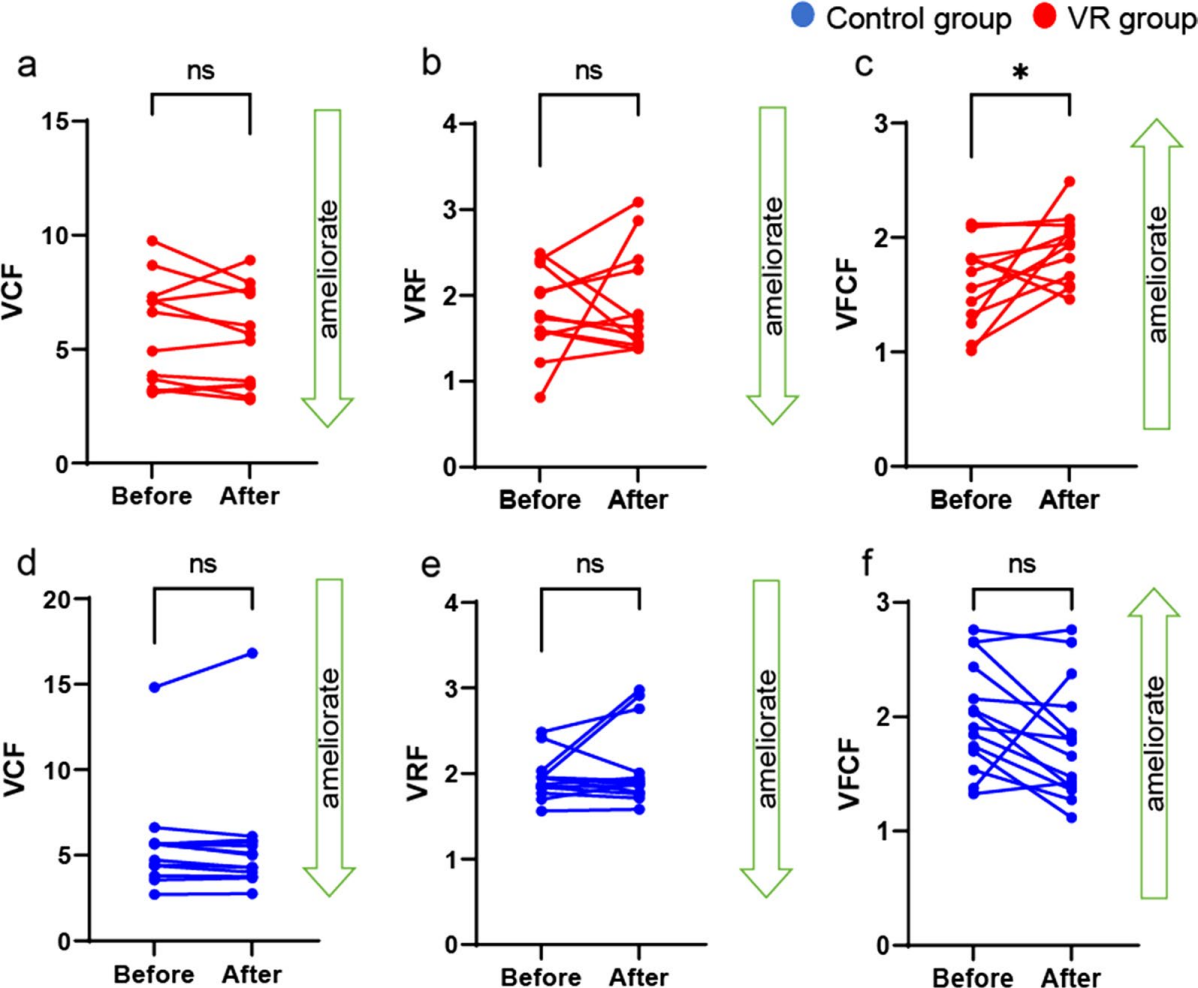
Static postural stability after mediVR KAGURA-guided training. **a**, **d** Changes in control group and VR group VCF parameters related to vestibular weighting: *P* = 0.96 (*r* = 0.13), *P* = 0.16 *(r* = 0.12), respectively. **b**, **e** Changes in control group and VR group VRF parameters related to visual weighting: *P* = 0.67 (*r* = 0.12). *P* = 0.31 (*r* = 0.15), respectively. **c**, **f** Changes in control group and VR group VFCF parameters related to somatosensory weighting: *P* = 0.04 (*r* = 0.53), *P* = 0.02 *(r* = 0.58), respectively. Blue indicates the control group, and red indicates the VR group. Bars indicate the mean ± SD. *VR* virtual reality, *VCF* velocity of COP in the eyes-closed/foam rubber condition, *VRF* velocity of Romberg’s ratio with foam rubber, *VFCF* velocity of foam ratio in the eyes-closed/foam rubber condition, *SD *standard deviation

To examine changes in static postural stability after mediVR KAGURA-guided training in patients with PPPD, we assessed changes in the parameters of vestibular weighting (VCF), visual weighting (VRF), and somatosensory weighting (VFCF) before and after training. The VR group demonstrated a significant improvement in VFCF after mediVR KAGURA-guided training compared to that before training (Fig. 2a–c, VCF: *P* = 0.16, VRF: *P* = 0.31, VFCF: *P* = 0.02 [Cohen’s d = 1.41]), suggesting somatosensory re-weighting in the vestibular control system. While no such improvement was observed in the control group (Fig. 2d–f, VCF: *P* = 0.98, VRF: *P* = 0.30, VFCF: *P* = 0.08).

### Dynamic postural stability was partly improved by mediVR KAGURA-guided training in patients with PPPD

**Fig. 3:**
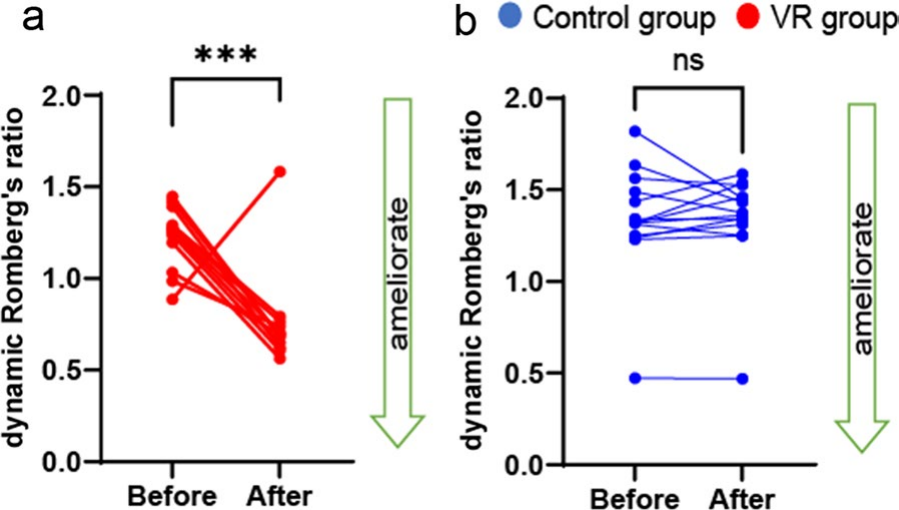
Dynamic postural stability after mediVR KAGURA-guided training. **a**, **b** Dynamic Romberg’s ratios in the (**a**) control group (*P* = 0.34, *r* = 0.12) and (**b**) VR group (*P* = 0.003, *r* = 0.80). Blue indicates the control group, and red indicates the VR group. Bars indicate the mean ± SD. *VR* virtual reality, *SD* standard deviation

To examine changes in dynamic postural stability after mediVR KAGURA-guided training in patients with PPPD, we compared changes in Foulage test [45, 46, 48]. The VR group exhibited significant improvement in the dynamic Romberg’s ratio, while no such improvement was observed in the control group (Fig. 3a, VR group: *P* = 0.003 [Cohen’s d = 2.42]; Fig. 3b, control group: *P* = 0.34), which suggests visual re-weighting or desensitization.

### Subjective symptoms and anxiety related to PPPD improved after mediVR KAGURA-guided training

**Fig. 4:**
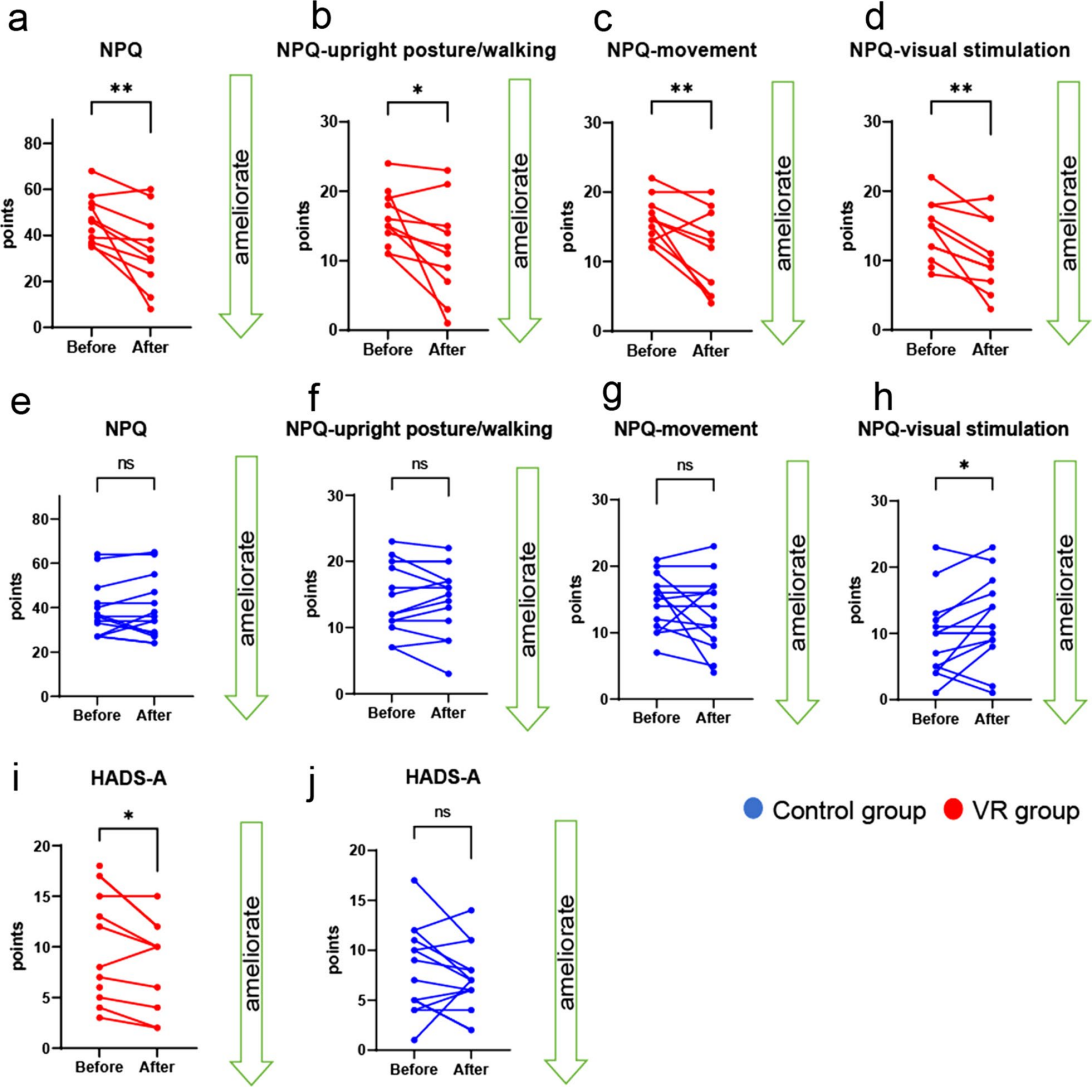
Subjective symptoms of PPPD after mediVR KAGURA-guided training and conventional physiotherapy. **a**–**d** Control group: NPQ: *P* = 0.40, *r* = 0.20 (**a**), NPQ upright posture/walking: *P* = 0.32, *r* = 0.23 (**b**), NPQ movement: *P* = 0.12, *r* = 0.30 (**c**), and NPQ visual stimulation: *P* = 0.02, *r* = 0.52 (**d**). **e**–**h** VR group: NPQ: *P* = 0.005, *r* = 0.74 (**e**), NPQ upright posture/walking: *P* = 0.02, *r* = 0.65 (**f**), NPQ movement: *P* = 0.005, *r* = 0.72 (**g**), and NPQ visual stimulation: *P* = 0.002, *r* = 0.77 (**h**). **i**, **j** HADS-A: *P* = 0.15, *r* = 0.24 in the control group and *P* = 0.01, *r* = 0.67 in the VR group. Blue indicates the control group, and red indicates the VR group. Bars indicate the mean ± SD. *PPPD* persistent postural-perceptual dizziness, *VR* virtual reality, *NPQ* Niigata PPPD Questionnaire, *HADS* Hospital Anxiety and Depression Scale, *SD* standard deviation

Responses to clinical questionnaires related to vestibular disorders were analyzed at baseline and after the mediVR KAGURA-guided training to examine changes in subjective symptoms. In the VR group, significant improvements in scores for the HADS-A, NPQ, and all NPQ subcategories relative to baseline were observed following mediVR KAGURA-guided training (Fig. 4a–d, i, NPQ: *P* = 0.005 [Cohen’s d = 2.20], NPQ upright posture/walking: *P* = 0.02 [Cohen’s d = 1.67], NPQ movement: *P* = 0.005 [Cohen’s d = 2.06], NPQ visual stimulation: *P* = 0.002 [Cohen’s d = 2.42], HADS-A: *P* = 0.01 [Cohen’s d = 1.78]). There were no significant differences in other parameters between baseline and after mediVR KAGURA-guided training (DHI: *P* = 0.08, DHI-P: *P* = 0.18, DHI-E: *P* = 0.06, DHI-F: *P* = 0.16, HADS-D: *P* = 0.24, OD: *P* = 0.17, PSQI: *P* = 0.50, Graybiel’s motion sickness score: *P* = 0.31).

In the control group, there were no significant differences in any parameter except for the deterioration of visual stimulation in the NPQ (Fig. 4e–h, j, NPQ: *P* = 0.43, NPQ upright posture/walking: *P* = 0.20, NPQ movement: *P* = 0.13, NPQ visual stimulation: *P* = 0.02 [Cohen’s d = 1.27], HADS-A: *P* = 0.19, DHI: *P* = 0.07, DHI-P: *P* = 0.38, DHI-E: *P* = 0.10, DHI-F: *P* = 0.17, HADS-D: *P* = 0.24, OD: *P* = 0.37, PSQI: *P* = 0.32, Graybiel’s motion sickness score: *P* = 0.06).

## Discussion

In this study, we aimed to determine the effectiveness of VR-guided training for the management of PPPD using the mediVR KAGURA system. Patients with PPPD exhibited improvements in objective outcomes, including static and dynamic postural stability (somatosensory and visual re-weighting), immediately after the mediVR KAGURA-guided training session. Additionally, this training improved anxiety and scores for all NPQ subcategories after 1 week. Consequently, these findings broaden the possibilities of PPPD management using mediVR KAGURA-guided rehabilitation.

PPPD is a syndrome that unifies the key features of PPV, CSD, and other related disorders [2]. Research has demonstrated that the mechanisms underlying PPPD include the stiffening of postural control, a shift in spatial orientation information processing to favor visual over vestibular inputs, and failure of higher-order cortical mechanisms to modulate the first two processes [1, 4–9]. Psychological and functional comorbidities develop from maladaptive cognitive-behavioral responses, including acrophobia, anxiety or depressive disorders, and functional gait abnormalities. Therefore, current strategies for managing PPPD include therapy for comorbidities such as vestibular diseases, re-weighting of sensory inputs for postural control [13–15], and desensitization to increase tolerance to perceived stimuli [16–18].

In this study, mediVR KAGURA-guided training resulted in improvements in NPQ scores, static postural stability (somatosensory weighting), dynamic postural stability (visual weighting), and even short-term rehabilitation. We speculate that re-tuning, desensitization, and re-weighting attenuated impairments in balance control by improving cognitive and motor function in patients with PPPD. The use of conventional approaches for quantitative assessments of performance during postural balance training may be ineffective for PPPD [26, 29]. VR creates multiple interactive, multimodal sensory stimuli that offer unique advantages over other approaches in neurorehabilitation. VR facilitates an easy, prompt, quantitative, and replicable training method by accurately setting the distance, angle, height, or other parameters in a 3D virtual space [26, 29]. The therapist’s only responsibility is to adjust these parameters by inputting numbers on the operating panel of the computer. Moreover, the randomly programmed VR-training tasks may require significantly more precise postural control. Consequently, the tailor-made, optimized, and unpredictable mediVR KAGURA-guided tasks can result in greater improvements in postural and trunk balance when compared with conventional approaches, even when performed in a sitting position. Previous research has indicated that the use of VR during neurorehabilitation improves cognitive and motor function [21], with several studies reporting benefits in relation to upper limb function, balance, and gait [51]; neuromotor monitoring of recovery [52]; strength fitness and skills [53], range of motion for the shoulder, and spasticity [54]. In this study, mediVR KAGURA-guided training promoted sensory re-weighting in patients with PPPD, resulting in improvement of subjective symptoms. This finding suggests that re-weighting of somatosensory inputs and desensitization to visual inputs via mediVR KAGURA-guided training are important for symptom improvement in patients with PPPD. We speculated that adequate and optimized VR-guided rehabilitation can have a greater effect on PPPD.

Our findings also indicated that anxiety scores improved following mediVR KAGURA-guided training when compared with those observed in the control group. This outcome highlights the benefits of using tailor-made, optimized, and unpredictable mediVR KAGURA-guided training tasks and therapist-guided relaxation techniques. During our mediVR KAGURA-guided rehabilitation sessions, the patient was constantly required to think of the next task to time the fall of the next object and perform the reaching movements in a timely manner, emulating the conditions necessary for hazard perception and avoidance. These methods may be useful for patients with PPPD who are unable to imagine the variables associated with real-world situations. A previous study reported psychological and cognitive benefits when older adults utilized adapted VR devices, such as improvements in attention or memory and decreased depressive symptoms [21, 55]. Taken together, the available evidence indicates that VR devices can be used as effective tools to motivate patients during rehabilitation sessions [56], to improve spatial orientation and attention in daily life activities [57], and to improve pain relief scores and emotional aspects related to functionality [55]. Additionally, a VR training environment can provide participants with task-specific training, accurate sensory and tactile feedback, and motivation [58]. Therefore, mediVR KAGURA dual-task training may facilitate neurorehabilitation of cognitive and motor function.

Notably, the application of VR technology in the future may benefit from a larger pool of automated data and advancements in artificial intelligence. For example, eye trackers may become a standard feature of VR headsets; this will allow for the collection of data from both healthy controls and individuals with disabilities. Given the potential for this to ensure equity in therapeutic settings despite differences in ability, further studies are required to explore this issue.

## Limitations

This study had several limitations. First, the mediVR KAGURA-guided training session was short (100 tasks for 10 min) because we could not use the machine for a longer duration due to funding limitations. Consequently, adjustments to the settings and treatment protocol were not attempted, although research indicates that longer interventions are more effective [23]. Second, the long-term effects on PPPD were not investigated. Further randomized and long-term studies comparing mediVR KAGURA-guided training and other rehabilitation programs are required to validate the effectiveness of this VR-guided method. Lastly, the sample size was insufficient for some analyses. Nonetheless, the study provides proof of concept, supporting the need for additional studies with larger sample sizes.

## Conclusion

Our findings indicated that a single session of VR-guided, dual-task, trunk balance training using a mediVR KAGURA system improved static and dynamic balance ability, NPQ scores, and HADS-A scores in patients with PPPD compared to conventional physiotherapy. VR-guided training using mediVR KAGURA offers a safe and cost-effective solution for PPPD. As such, further prospective studies need to explore the adequate conditions for this VR-guided method.

## Supplementary Information

**Additional file 1.** Overview of the assessment tools used in this study and their respective scoring systems. Scoring and criteria of questionnaire and equilibrium test.

**Additional file 2.** Movie during mediVR-guided training. Motion during the VR-training.

## Data Availability

All data that support the findings of this study are available from the corresponding author, Toru Miwa, upon reasonable request.

## Abbreviations

PPPD: Persistent postural-perceptual dizziness
VR: Virtual reality
NPQ: Niigata PPPD Questionnaire
PPV: Phobic postural vertigo
CSD: Chronic subjective dizziness
DHI: Dizziness Handicap Inventory
HADS: Hospital Anxiety and Depression Scale
OD: Orthostatic dysregulation
PSQI: Pittsburgh Sleep Quality Index
COP: Center of pressure
VCF: Velocity of COP in the eyes-closed/foam rubber condition
VRF: Velocity of Romberg’s ratio with foam rubber
VFCF: Velocity of foam ratio in the eyes-closed/foam rubber condition
FT: Foulage test
SD: Standard deviation
