# Supplementary material for: Temporal virtual reality-guided, dual-task, trunk balance training in a sitting position improves persistent postural-perceptual dizziness: proof of concept
Source: J Neuroeng Rehabil. 2022 Aug 20;19:92. doi: 10.1186/s12984-022-01068-6 (PMC9392908; doi:10.1186/s12984-022-01068-6)
Supplement: Supplementary file 1 — Additional file 1. Overview of the assessment tools used in this study and their respective scoring systems. Scoring and criteria of questionnaire and equilibrium test. [file 12984_2022_1068_MOESM1_ESM.docx]

**Additional File 1**

**Supplementary Table:** Overview of the assessment tools used in this study and their respective scoring systems

|  | Aim | Test | Scoring and criteria |
| --- | --- | --- | --- |
| **Questionnaires** | Degree of vertigo/dizziness | DHI [34] | 100-point scale, 25 items  Sub-grouped into three domains: Physical (P), Emotional (E), Functional (F) |
|  | Anxiety and depression | HADS [36] | Anxiety (A) 21-point scale, 7 questions  Depression (D) 21-point scale, 7 questions |
|  | OD | OD questionnaire [38,39] | 11-point scale, 11 questions |
|  | Motion sickness | Graybiel’s motion sickness scores [40] | 56-point scale, 6 questions |
|  | Sleep quality | PSQI [41,42] | 21-point scale, 18 questions |
| **Tests** | Static postural stability | Foam Stabilometry [44] | VCF (Vestibular weighting)  VRF (Visual weighting)  VFCF (Somatosensory weighting)  Small VCF and VRF and large VFCF indicate improvement in PPPD |
|  | Dynamic postural stability | Foulage test; stepping test [45–48] | Dynamic Romberg’s ratio (FT values eyes-closed/eyes-open)  Romberg’s ratios indicate PPPD improvement |

Abbreviations: DHI: Dizziness Handicap Inventory; HADS: Hospital Anxiety and Depression Scale; OD: orthostatic dysregulation; PSQI: Pittsburgh Sleep Quality Index; COP: center of pressure; VCF: velocity of movement of the COP in eyes-closed/foam rubber condition; VRF: velocity of Romberg's ratios with foam rubber; VFCF: velocity of foam ratios (without/with the foam rubber) in the eyes-closed condition; FT value: Foulage test value; PPPD: persistent postural-perceptual dizziness.
